# Mitral regurgitation quantified by CMR 4D-flow is associated with microvascular obstruction post reperfused ST-segment elevation myocardial infarction

**DOI:** 10.1186/s13104-022-06063-7

**Published:** 2022-05-15

**Authors:** Hosamadin Assadi, Ciaran Grafton-Clarke, Ahmet Demirkiran, Rob J. van der Geest, Robin Nijveldt, Marcus Flather, Andrew J. Swift, Vass S. Vassiliou, Peter P. Swoboda, Amardeep Dastidar, John P. Greenwood, Sven Plein, Pankaj Garg

**Affiliations:** 1 Department of Cardiovascular and Metabolic Health, Norwich Medical School, University of East Anglia, Colney Lane, Norwich, NR4 7UQ UK; 2 Department of Cardiology, Norfolk and Norwich University Hospital, Norwich, UK; 3 Department of Cardiology, Amsterdam UMC, Vrije Universiteit Amsterdam, Amsterdam Cardiovascular Sciences, Amsterdam, The Netherlands; 4 Division of Image Processing, Department of Radiology, Leiden University Medical Center, Leiden, The Netherlands; 5 Department of Cardiology, Radboud University Medical Center, Nijmegen, The Netherlands; 6 Department of Infection, Immunity and Cardiovascular Disease, University of Sheffield, Sheffield, UK; 7 Multidisciplinary Cardiovascular Research Centre & Leeds Institute of Cardiovascular and Metabolic Medicine, University of Leeds, Leeds, UK; 8 Bristol Medical School, University of Bristol, Bristol, UK

**Keywords:** ST-segment elevation myocardial infarction, Magnetic resonance imaging, Haemodynamics, Mitral regurgitation

## Abstract

**Objectives:**

Mitral regurgitation (MR) and microvascular obstruction (MVO) are common complications of myocardial infarction (MI). This study aimed to investigate the association between MR in ST-elevation MI (STEMI) subjects with MVO post-reperfusion. STEMI subjects undergoing primary percutaneous intervention were enrolled. Cardiovascular magnetic resonance (CMR) imaging was performed within 48-hours of initial presentation. 4D flow images of CMR were analysed using a retrospective valve tracking technique to quantify MR volume, and late gadolinium enhancement images of CMR to assess MVO.

**Results:**

Among 69 patients in the study cohort, 41 had MVO (59%). Patients with MVO had lower left ventricular (LV) ejection fraction (EF) (42 ± 10% vs. 52 ± 8%, P < 0.01), higher end-systolic volume (98 ± 49 ml vs. 73 ± 28 ml, P < 0.001) and larger scar volume (26 ± 19% vs. 11 ± 9%, P < 0.001). Extent of MVO was associated with the degree of MR quantified by 4D flow (R = 0.54, P = 0.0003). In uni-variate regression analysis, investigating the association of CMR variables to the degree of acute MR, only the extent of MVO was associated (coefficient = 0.27, P = 0.001). The area under the curve for the presence of MVO was 0.66 (P = 0.01) for MR > 2.5 ml. We conclude that in patients with reperfused STEMI, the degree of acute MR is associated with the degree of MVO.

## Introduction

Post reperfused ST-elevation myocardial infarction (STEMI), the ventricles undergo structural adaptations both within and outside of the infarct, referred to as left ventricular (LV) remodelling. In some patients, coronary reperfusion is associated with microvascular obstruction (MVO), seen angiographically as no-reflow. MVO is associated with adverse prognosis, independently of infarct size (IS) [1, 2]. Clinically, the presence and the extent of MVO can be assessed by cardiovascular magnetic resonance imaging [3, 4].

In addition, mitral regurgitation (MR) of any degree is a powerful, independent predictor of mortality in patients undergoing primary percutaneous reperfusion therapy for STEMI. The presence of MR identifies high-risk patients in whom close out-patient follow-up is warranted, and who may benefit from aggressive adjunctive medical or surgical therapies [5–8]. CMR can be used for MR quantification [9]. More recently, four-dimensional (4D) flow imaging has demonstrated better precision for MR quantification than standard methods of MR quantification by CMR [10–12]. These advanced 4D flow CMR methods allow quantification of even a small degree of MR [13].

Even though both MVO and acute MR are associated with adverse outcomes post reperfused STEMI, it remains unknown if both are associated with each other.

We hypothesise that the extent of MVO after reperfused STEMI is associated with the degree of acute MR. Hence, the aim of this study was to investigate if the degree of MR is associated with the degree of MVO. In addition, we aimed to evaluate if the presence of MR can have diagnostic value for the presence of MVO.

## Main text

### Methods

#### Ethics approval and consent to participate

This study was conducted as per the principles outlined in the 1964 Declaration of Helsinki. The collection and management of data were approved by the National Research Ethics Service in the United Kingdom (12/YH/0169). Written informed consent was obtained from all individual participants in the study.

#### Study population

This was a prospective cohort study in which 69 patients with acute reperfused STEMI were enrolled. The patients were enrolled at the Leeds Teaching Hospitals (University of Leeds, Leeds, United Kingdom). All patients had CMR examination within 48-h of the index event.

Exclusion criteria were prior history of myocardial infarction or any coronary revascularisation procedure, non-ischemic cardiomyopathy, and any contraindication to CMR.

#### CMR protocol and analysis

CMR imaging was done on a 1.5T system (Ingenia, Philips, Best, The Netherlands) with a phased-array 28-channel cardiac receiver coil. The CMR protocol included conventional cine imaging, 4D flow and early/late gadolinium enhancement (EGE/LGE) imaging.

Cine imaging, LGE imaging, and 4D flow acquisition methods have been previously published by our group [14–18]. For 4D flow acquisition, three-dimensional, three-directional phase-contrast, and velocity encoded data were acquired within the LV using an Echo-Planar Imaging accelerated, free-breathing sequence with retrospective ECG-triggering. Gradient nonlinearity and Maxwell correction were automatically performed by the scanner. Typical acquisition parameters were: TE/TR 3.7 ms/11 ms, flip angle 10°, VENC 150 cm/s and voxel size 3.0 × 3.0 × 3.0 mm (30 phases per cardiac cycle).

All CMR analysis was performed using dedicated research software (MASS version 2021-Exp, Leiden University Medical Center, Leiden, the Netherlands). All CMR contour tracings, including volume/function, LGE, and the 4D flow CMR were performed by R.J.G. and controlled by EACVI level-III certified CMR experts (P.G. and R.N.), all with over 10 years of CMR experience.

Infarct size was calculated on the short-axis LGE images using the full-width-at-half-maximum method [14]. MVO was defined visually as the hypointense core within the infarcted zone and planimetered manually. Volumes of infarct and MVO were calculated from planimetered areas across the whole LV stack by the modified Simpson’s method [1, 2].

#### Mitral inflow analysis

Mitral inflow analysis was performed on the 4D flow CMR images. The mitral valve position was determined on the LV two- and four-chamber cine acquisitions. Manual retrospective valve tracking over the mitral valve was performed. The planes of flow quantification followed the valve plane over the cardiac cycle and were adjusted according to the blood flow direction. On the reformatted valve plane phase-contrast images, the mitral inflow was identified as higher signal intensity during LV diastole and segmented appropriately throughout the complete cardiac cycle to obtain the trans-mitral flow curve. The mitral regurgitation jet was identified during the left ventricular systolic phases. The reformatted plane during systole was planned perpendicular to the MR jet in the left atrium to capture the jet [9]. Mitral valve stroke volume was computed by the following formula: mitral forward flow during left ventricular diastole—mitral backward flow during systole.

### Statistical analysis

Statistical analysis was performed using MedCalc (Version 20.011). Continuous variables are presented as mean ± standard deviation or median with interquartile range, as appropriate. Comparisons between two groups were made with the independent-samples T-test for normally distributed data or Mann‐Whitney U test for non‐parametric data. Categorical variables are summarised by frequency (percentage), and relationships between categorical variables were tested with the χ^2^ test. Association between continuous variables was quantified by Pearson’s correlation. Kruskal–Wallis test was used for one-way ANOVA testing on non-parametric data. Receiver-operating characteristics (ROC) analysis was performed to assess the performance of MR to predict the presence of MVO. Areas under the ROC curve were calculated, and Youden’s index was used to determine optimal cut-off values. Multivariate linear regression was performed using Enter and Stepwise procedures to evaluate independent association to the extent of MVO. The two-sided significance level was set at 5%.

### Results

#### Patient population

Baseline characteristics of the study population (n = 69) are summarised in Table 1. Forty-one patients had MVO (59%). Patients with MVO had larger left ventricular volumes and significantly lower left ventricular ejection fraction. Patients with MVO also had higher scar volume (26 ± 19% vs. 11 ± 9%, P < 0.001).

**Table 1:** Patient demographics

	MVO−ve	MVO+ve	P-value
	n = 28	n = 41	
Age (years)	62 ± 13	62 ± 11	0.935
Sex (male)	6 (21)	8 (20)	0.945
Weight (kg)	79 ± 17	83 ± 16	0.321
Height (cm)	172 ± 10	172 ± 8	0.918
BMI	27 ± 5	29 ± 4	0.222
Smoker	23 (79)	18 (45)	0.049
Hypertension	9 (31)	12 (30)	0.392
Hypercholesterolaemia	8 (28)	11 (28)	0.524
Diabetes mellitus	4 (14)	5 (13)	0.877
Family history of CAD	15 (52)	16 (40)	0.91
LVEDV (ml)^a^	147 ± 50	183 ± 48	0.004
LVESV (ml)^a^	73 ± 28	98 ± 49	< 0.001
LVSV (ml)	81 ± 16	73 ± 17	0.053
LV EF (%)	52 ± 8	42 ± 10	< 0.01
LV mass (g)	117 ± 29	129 ± 33	0.13
Scar (% of LV)^a^	11 ± 9	26 ± 19	< 0.001
4D flow mitral SV (ml)^a^	66 ± 26	55 ± 15	0.002
4D flow MR (ml)^a^	2.4 ± 4	4.2 ± 4	0.02

*4D* four-dimensions, *BMI* body mass index, *CAD* coronary artery disease, *EF* ejection fraction, *LV* left ventricle, *LVEDV* left ventricular end-diastolic volume, *LVESV* left ventricular end-systolic volume, *LVSV* left ventricular stroke volume, *SV* stroke volume, *MR* mitral regurgitation

^a^Non-parametric data (median ± IQR)

#### Association of standard CMR variables to MVO

The degree of MVO was associated with all volumetric parameters including left ventricular end-diastolic volume (r = 0.33, P = 0.006), end-systolic volume (r = 0.52, P < 0.001), stroke volume (r = − 0.35, P = 0.003), ejection fraction (r = − 0.59, P < 0.001) and mass (r = 0.4, P = 0.003). In addition, the degree of left ventricle scar was also linearly associated with the degree of MVO (r = 0.58, P < 0.001).

#### 4D flow CMR analysis

**Fig. 1:**
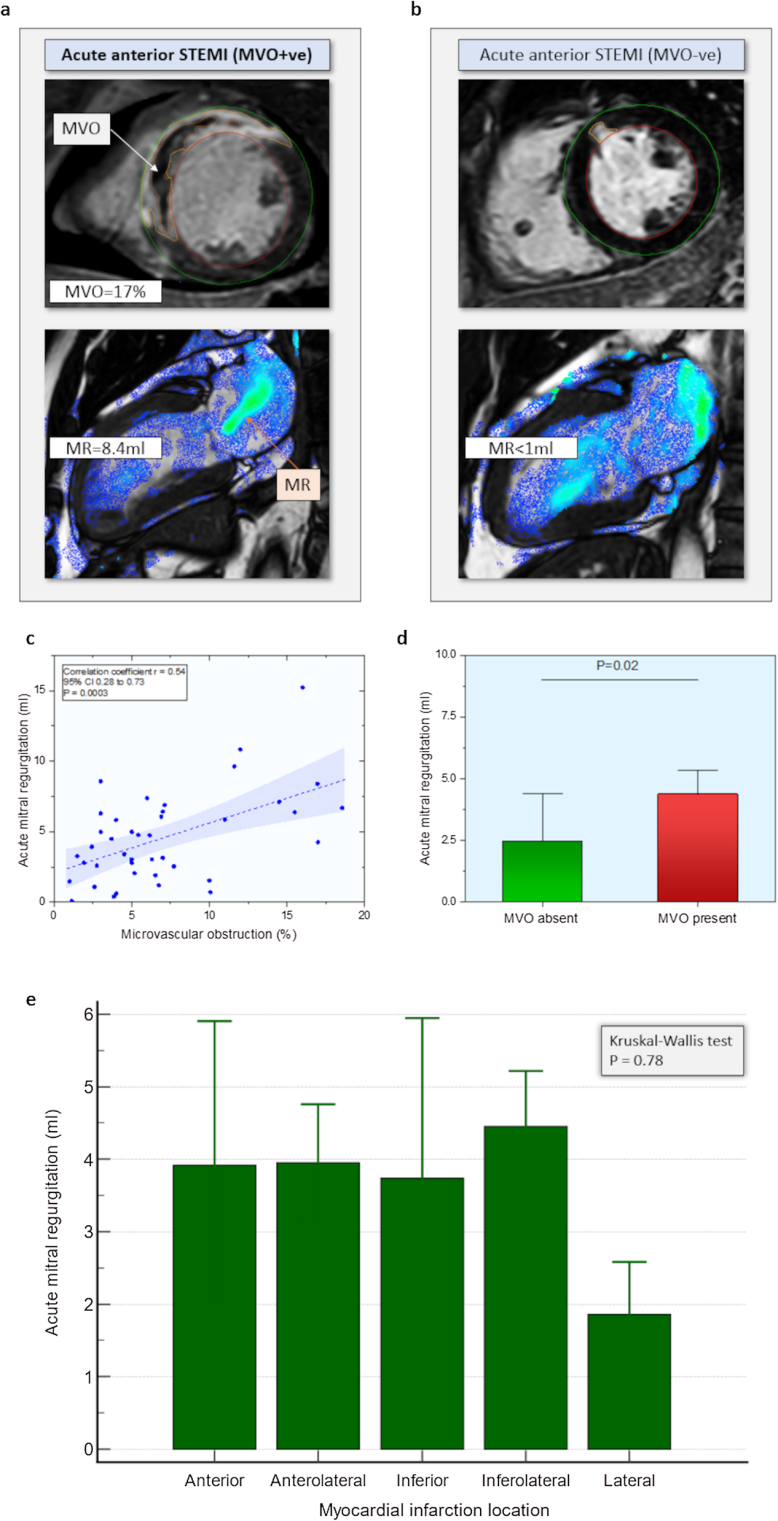
**a** A case example from the study with a large anterior STEMI with microvascular obstruction and central mitral regurgitation jet with MR volume of 8.4 ml. **b** A case example of a study demonstrating a small infarct with very little evidence of mitral regurgitation. **c** Scatter plot demonstrating an association between the degree of acute MR and MVO. **d** Bar chart demonstrating differences in acute MR and with/without the presence of MVO (median and its standard error, P-value Mann–Whitney). **e** Bar chart of the location of infarct and its association to the extent of acute MR

Mitral stroke volume was significantly reduced in MVO patients (55 ± 15 ml vs. 66 ± 26 m, P = 0.001). Degree of MVO demonstrated association with the degree of MR quantified by 4D flow (Pearson correlation = 0.54, 95% CI 0.28–0.73, P = 0.0003) (Fig. 1a–d). The area under the curve for the prediction of MVO by the extent of MR was 0.66 (P = 0.01). An MR greater than 2.5 ml demonstrated 75% sensitivity and 57% specificity for the diagnosis of MVO.

#### Association of MR to infarct territory

In the whole cohort, 37 cases (53%) were anterior infarcts and 21 (30%) were inferior infarcts. Rest 17% were either lateral, anterolateral or inferolateral. The volume of MR was not different in either of these categories (Fig. 1e, P = 0.78).

#### Independent predictors of MVO on uni/multi-variate regression

In univariate regression analysis, all CMR volumetric and tissue characterisation variables demonstrated association to the extent of MVO (Table 2). Using the ‘Stepwise’ multiple linear regression, four CMR variables demonstrated independent association to the extent of MVO: the degree of mitral regurgitation (coefficient 0.44, P = 0.001), left ventricular ejection fraction (coefficient − 0.17, P = 0.002), left ventricular end-diastolic mass (coefficient 0.03, P = 0.04) and the extent of LV scar (coefficient 0.12, P = 0.002).

**Table 2:** Univariate and multi-variate regression of extent of acute MR and MVO to other CMR variables

Independent variables	Univariate			Multivariate		
	Beta	SE	P	Beta	SE	P
Association to extent of MVO						
LV end-diastolic volume (ml)	0.04	0.02	0.01	–	–	–
LV end-systolic volume (ml)	0.08	0.02	< 0.0001	–	–	–
LV stroke volume (ml)	− 0.11	0.03	0.00	–	–	–
LV ejection fraction (%)	− 0.29	0.05	< 0.0001	− 0.17	0.05	0.002
LV end-diastolic mass (g)	0.05	0.02	0.02	0.03	0.01	0.041
Mitral valve stroke volume (ml)	− 0.10	0.03	0.00			
LV scar (%)	0.22	0.04	< 0.0001	0.12	0.04	0.002
Acute MR (ml)	0.60	0.17	0.00	0.44	0.13	0.001
Association to extent of MR						
LV end-diastolic volume (ml)	0.01	0.01	0.27	–	–	–
LV end-systolic volume (ml)	0.02	0.01	0.14	–	–	–
LV stroke volume (ml)	− 0.02	0.02	0.53	–	–	–
LV ejection fraction (%)	− 0.04	0.04	0.36	–	–	–
LV end-diastolic mass (gram)	0.01	0.01	0.58	–	–	–
Mitral valve stroke volume (ml)	− 0.04	0.02	0.09	–	–	–
LV scar (%)	0.04	0.03	0.15	–	–	–
MVO (%)	0.27	0.07	0.001	–	–	–

*ml* millilitres, *SE* standard error, *LV* left ventricle, *CMR* cardiovascular magnetic resonance, *MR* mitral regurgitation, *MVO* microvascular obstruction

#### Independent predictors of acute MR on uni/multi-variate regression

On the contrary, in univariate regression analysis for investigating association to the degree of acute MR, only the extent of MVO was associated (coefficient 0.27, P = 0.001).

### Discussion

In this study, we report that in reperfused STEMI patients, the extent of acute MR is independently associated with MVO. It is well established that after acute reperfusion therapy in STEMI, both MVO and MR are associated with poorer outcomes [7, 19] and adverse remodelling, leading to heart failure [20, 21]. Importantly, the anatomical and physio-pathological mechanisms from which the acute MR originates have not been fully clarified. For the first time in this study, we have noted the observation that the degree of acute MR immediately after primary percutaneous coronary intervention (PPCI) is independently associated with the extent of MVO.

Prior studies have noted the association between papillary muscle infarction (PapMI) and MR with MVO in patients with STEMI. Lei et al*.* [22] recruited 209 patients from two centres and demonstrated that patients with PapMI and MR had more MVO when compared with non-PapMI and PapMI only groups (P = 0.004). These results further support the findings of our study. In an observational study done by Amigoni et al*.* [20], which recruited 496 patients, the authors demonstrated that almost 50% of patients did not have any acute MR on colour Doppler, whereas we were able to detect and quantify trivial MR by 4D flow CMR in 100% of cases. In their work, only 10% of patients had moderate MR, and a very limited number of patients had severe MR (2.8%), versus, in our smaller cohort, where none of the patients had moderate or severe MR. We suspect that these observations could be because our study is more contemporary, and PPCI services have significantly evolved since 2006, resulting in lesser ischaemic functional MR. They noted that the degree of MR was associated with ventricular dilatation, whereas we did not see this association. This discrepancy can again be explained by lack of moderate to severe MR in our cohort.

Noting that MVO is associated with larger infarcts and adverse remodelling [21], one could infer from our study that even quantification of milder acute MR is relevant. This is further substantiated by a study that investigated the survival of patients with or without ischaemic MR on colour Doppler and demonstrated that any MR had a significantly worse outcome (log-rank 3.8, P = 0.05) [23].

Historic study data suggests that ischaemic MR is more seen in inferior MI (61%) versus anterior MI (32%) [24]. However, this is still debated as the data was mainly observational and of a sub-selected patients’ cohort with chronic ischaemic MR going for mitral valve surgery versus acute MR. Our study demonstrates that in patients with trivial to mild acute MR, there remains no association with the location of myocardial infarction. Moreover, the degree of acute MR in this study was not directly associated with any volumetric changes, suggesting that MVO alone, which is a result of a prolonged period of myocardial ischaemia, has a specific pathophysiological process that may be associated with the development of acute MR post-STEMI.

Future studies are warranted to develop a better mechanistic understanding of acute MR in patients with MVO post-reperfusion therapy. In addition, a direct comparison of colour Doppler echocardiography to 4D flow CMR quantified MR is warranted to understand how much MR is needed to be detected by Doppler echocardiography for clinical translation between these two cardiac imaging modalities.

### Conclusions

In patients with reperfused STEMI, the degree of acute MR was associated with the degree of MVO. Future studies are warranted to develop insight into the pathophysiological process of how MVO is associated with acute MR.

## Limitations

Our study has some limitations. The results of our research is hypothesis-generating. Our study results cannot be generalised to all acute myocardial infarction patients. This study did not recruit hemodynamically unstable patients, which potentially introduces selection bias. Additionally, we did not have echocardiographic findings recorded in the study for demonstrating clinical translation between CMR and echocardiography. Prior MR was not exclusion criteria, and we assumed that MR noted on CMR was arising due to the acute myocardial infarction. Finally, this study did not explore the association of other clinical characteristics of acute myocardial infarction presentation and MVO as the sample size was small.

## Data Availability

Underlying data: access to the raw images of patients is not permitted since specialised post-processing imaging-based solutions can identify the study patients in the future.

## Abbreviations

4D: Four-dimensional
CMR: Cardiovascular magnetic resonance
EGE: Early Gadolinium Enhancement
IS: Infarct size
LGE: Late gadolinium enhancement
LV: Left ventricle
MR: Mitral regurgitation
MVO: Microvascular obstruction
MI: Myocardial infarction
PPCI: Primary percutaneous coronary intervention
ROC: Receiver-operating characteristics
STEMI: ST-elevation myocardial infarction
